# Association of Anthropometric and Bioelectrical Impedance Analysis Measures of Adiposity with High Molecular Weight Adiponectin Concentration

**DOI:** 10.1371/journal.pone.0156041

**Published:** 2016-05-26

**Authors:** Wei-Fang Zeng, Yan Li, Chang-Sheng Sheng, Qi-Fang Huang, Yuan-Yuan Kang, Lu Zhang, Shuai Wang, Yi-Bang Cheng, Fei-Ka Li, Ji-Guang Wang

**Affiliations:** Centre for Epidemiological Studies and Clinical Trials, Shanghai Key Laboratory of Hypertension, The Shanghai Institute of Hypertension, Department of Hypertension, Ruijin Hospital, Shanghai Jiaotong University School of Medicine, Shanghai, China; Tulane School of Public Health and Tropical Medicine, UNITED STATES

## Abstract

**Objective:**

To investigate the relationship between adiposity measures and plasma concentration of high molecular weight (HMW) adiponectin.

**Methods:**

In a Chinese sample (n = 1081), we performed measurements of anthropometry and bioelectrical impedance analysis (BIA). We defined overweight and obesity as a body mass index between 24 and 27.4 kg/m² and ≥ 27.5 kg/m², respectively, and central obesity as a waist circumference ≥ 90 cm in men and ≥ 80 cm in women. Plasma HMW adiponectin concentration was measured by the ELISA method.

**Results:**

Plasma HMW adiponectin concentration was significantly (*P* < 0.0001) higher in women (n = 677, 2.47 μg/mL) than men (n = 404, 1.58 μg/mL) and correlated with advancing age in men (r = 0.28) and women (r = 0.29). In adjusted analyses, it was lower in the presence of overweight (n = 159, 1.26 μg/mL in men and n = 227, 2.15μg/mL in women) and obesity (n = 60, 1.31 μg/mL and n = 82, 2.10 μg/mL, respectively) than normal weight subjects (n = 185, 2.07μg/mL and n = 368, 2.94 μg/mL, respectively) and in the presence of central obesity (n = 106, 1.28 μg/mL and n = 331, 2.12 μg/mL, respectively) than subjects with a normal waist circumference (n = 298, 1.74 μg/mL and n = 346, 2.74 μg/mL, respectively). In multiple regression analyses stratified for gender, adjusted for confounders and considered separately each of the adiposity measures, all adiposity measures were significantly (r -0.18 to -0.31, *P* < 0.001) associated with plasma HMW adiponectin concentration. However, in further stratified and adjusted regression analyses considered stepwise all adiposity measures, only waist-to-hip ratio was significantly (*P* < 0.05) associated with plasma HMW adiponectin concentration in men (r = -0.10) and women (r = -0.15).

**Conclusions:**

Anthropometric measures of obesity, such as waist-to-hip ratio, but not BIA measures, are independently associated with plasma adiponectin concentration.

## Introduction

Adiponectin is mostly expressed in the adipose tissue [1], and secreted into the circulation as a trimer or hexamer or as a high molecular weight (HMW) form [2]. Adiponectin is primarily an insulin-sensitizer [3], but it also has been shown to have anti-inflammatory and anti-atherogenic properties [4]. The HMW adiponectin is the most bioactive form, and has been found to be associated with endothelial dysfunction [4], hyperinsulinemia [5], type 2 diabetes mellitus [5] and other metabolic disorders [6,7]. The secretion of adiponectin is powerfully inhibited by fat accumulation, especially visceral fat accumulation [8]. Adiponectin is closely related to obesity and recognized as a critical link between obesity and obesity-related diseases, such as hypertension [9,10], type 2 diabetes mellitus [11,12], and the metabolic syndrome [13,14].

Obesity can be evaluated by the use of anthropometry, bioelectrical impedance analyses or computed tomography (CT) and magnetic resonance (MR) imaging [15]. Anthropometric measurements are the most often used method for the definition and classification of obesity in clinical practice and epidemiological settings. Body mass index, calculated as the body weight in kilograms divided by the body height in meters squared, is used to define overweight and obesity. Waist circumference or the ratio of waist-to-hip circumference is used to define central obesity or visceral obesity. These methods are useful, but often criticized for inaccuracy [16], especially when compared with the CT or MR imaging method [15]. However, the latter method has apparent limitations, for instance, cost. Devices that operate bioelectrical impedance analysis (BIA) technique are therefore developed and become popular in the evaluation of obesity, especially central obesity in the clinical setting and at home [17,18].

In the present study, we investigated in a Chinese population sample associations of anthropometric and BIA measures of adiposity with plasma HMW adiponectin concentration.

## Materials and Methods

### Study Population

The present cross-sectional analysis was based on data of an ongoing population study on multiple cardiovascular risk factors in Shanghai, China [19–21]. The study subjects were recruited from a newly established residential area in the suburb of Shanghai, 30 kilometers from the city center. Most residents were immigrants from nearby villages since 2003 and previously doing farming or other agricultural work. The Ethics Committee of Ruijin Hospital, Shanghai Jiaotong University School of Medicine approved the study protocol. All subjects gave written informed consent.

In the year 2009, we invited 1630 residents to take part in our study. Of those invited, 1192 (73.1%) participated. We excluded 111 subjects from the present analysis because they did not collect blood samples (n = 67) or anthropometric data (n = 70). Thus, the total number of subjects included in the present analysis was 1081.

### Field Work

One experienced physician administered a standardized questionnaire to collect information on medical history, lifestyle and use of medications. A trained technician performed anthropometric measurements. Body height was measured to the nearest 0.5 cm, and body weight with light indoor clothes and without shoes. Body mass index was calculated as the body weight in kilograms divided by the body height in meters squared. Waist and hip circumferences were measured to the nearest 0.5 cm at the narrowest part of the torso and the widest portion of the buttocks, respectively. Waist-to-hip ratio is the circumference of the waist divided by that of the hip. We performed BIA to measure body fat percentage, visceral fat percentage, and visceral-body fat ratio using the validated four-limb HBF359 device (Omron, Tokyo, Japan) [17]. We defined overweight and obesity as a body mass index between 24 and 27.4 kg/m² and ≥ 27.5 kg/m², respectively, and central obesity as a waist circumference of at least 90 cm in men and 80 cm in women.

### Laboratory Methods

Venous blood samples were drawn after overnight fasting for biochemical measurements. Plasma HMW adiponectin concentration was measured by the ELISA method (R&D Systems, Inc., Minneapolis, MN). The within-assay and between-assay coefficients of variation were 5.0% and 7.3%, respectively. Plasma glucose concentration was measured by a clinical chemistry automatic analyzer (Hitachi 7600–020, Tokyo, Japan).

Hypoadiponectinemia was defined as a plasma HMW adiponectin concentration within the gender-specific bottom decile. Diabetes mellitus was defined as a plasma glucose concentration of at least 7.0 mmol/L fasting or 11.1 mmol/L at any time, or as using antidiabetic agents. Prediabetes was defined as a fasting plasma glucose concentration in the range of 6.1 to 6.9 mmol/L.

### Statistical Analysis

For database management and statistical analysis, we used SAS software (version 9.13, SAS Institute, Cary, NC, USA). Means and proportions were compared with the Student *t*-test and Fisher’s exact test, respectively. For statistical analysis, plasma HMW adiponectin concentration was logarithmically transformed. We performed analyses of covariance for the comparison of plasma HMW-adiponectin concentration between various subgroups according to gender, age and obesity status and for the computation of adjusted mean values of plasma HMW-adiponectin concentration, while controlling for confounding factors. We performed multiple regression analyses to study associations of plasma HMW-adiponectin concentration with anthropometric and BIA measures of obesity in a full model with each of the adiposity measures considered separately and in a stepwise model with all adiposity measures considered simultaneously. We performed multiple logistic regression analyses to study associations between waist-to-hip ratio and hypoadiponectinemia.

## Results

The 1081 participants included 404 (37.4%) men, and 96 (8.9%) diabetic and prediabetic patients, of whom 48 (4.4%) used antidiabetic drugs. Men and women differed significantly in all characteristics (*P* ≤ 0.03 for the significant characteristics) except for the prevalence of diabetes or prediabetes and the use of antihypertensive or antidiabetic drugs (*P* ≥ 0.21, **Table 1**).

**Table 1:** Characteristics of the study population.

Characteristic	Men (n = 404)	Women (n = 677)	*P* value
**Age, years**	**56.7 ± 12.7**	**54.2 ± 13.2**	**0.004**
**Body height, cm**	**164.4 ± 6.4**	**154.2 ± 6.0**	**< 0.001**
**Body weight, kg**	**65.8 ± 10.5**	**56.6 ± 8.9**	**< 0.001**
**Body mass index, kg/m**^**2**^	**24.3 ± 3.3**	**23.8 ± 3.2**	**0.011**
**Body mass index ≥ 27.5 kg/m**^**2**^**, n (%)**	**60 (14.9)**	**82 (12.1)**	**0.226**
**Waist circumference, cm**	**83.7 ± 8.9**	**79.0 ± 8.6**	**< 0.001**
**Waist circumference ≥ 90 cm in men and ≥ 80 cm in women, n (%)**	**106 (26.2)**	**331 (48.9)**	**< 0.001**
**Hip circumference, cm**	**93.8 ± 6.2**	**92.9 ± 6.3**	**0.017**
**Waist-to-hip ratio**	**0.89 ± 0.05**	**0.85 ± 0.06**	**< 0.001**
**Body fat percentage, %**	**26.3 ± 4.7**	**34.1 ± 4.8**	**< 0.001**
**Visceral fat percentage, %**	**11.3 ± 4.7**	**7.1 ± 3.4**	**< 0.001**
**Visceral-body fat ratio**	**0.42 ± 0.14**	**0.20 ± 0.08**	**< 0.001**
**Hypertension, n (%)**	**170 (42.1)**	**238 (35.2)**	**0.027**
**Use of antihypertensive drugs, n (%)**	**130 (32.2)**	**193 (28.6)**	**0.217**
**Diabetes or prediabetes, n (%)**	**38 (9.4)**	**58 (8.6)**	**0.659**
**Use of antidiabetic drugs, n (%)**	**17 (4.2)**	**31 (4.6)**	**0.879**
**Current smoking, n (%)**	**198 (49.0)**	**3 (0.4)**	**< 0.001**
**Alcohol intake, n (%)**	**147 (36.4)**	**9 (1.3)**	**< 0.001**
**Plasma HMW adiponectin concentration, μg/mL**	**1.58 (1.47–1.71)**	**2.47 (2.33–2.61)**	**< 0.001**

HMW: High molecular weight. Values are arithmetic (± standard deviation) or geometric mean (95% confidence interval) or number of subjects (%). For definitions of hypertension, diabetes mellitus and prediabetes, see Methods.

**Fig 1:**
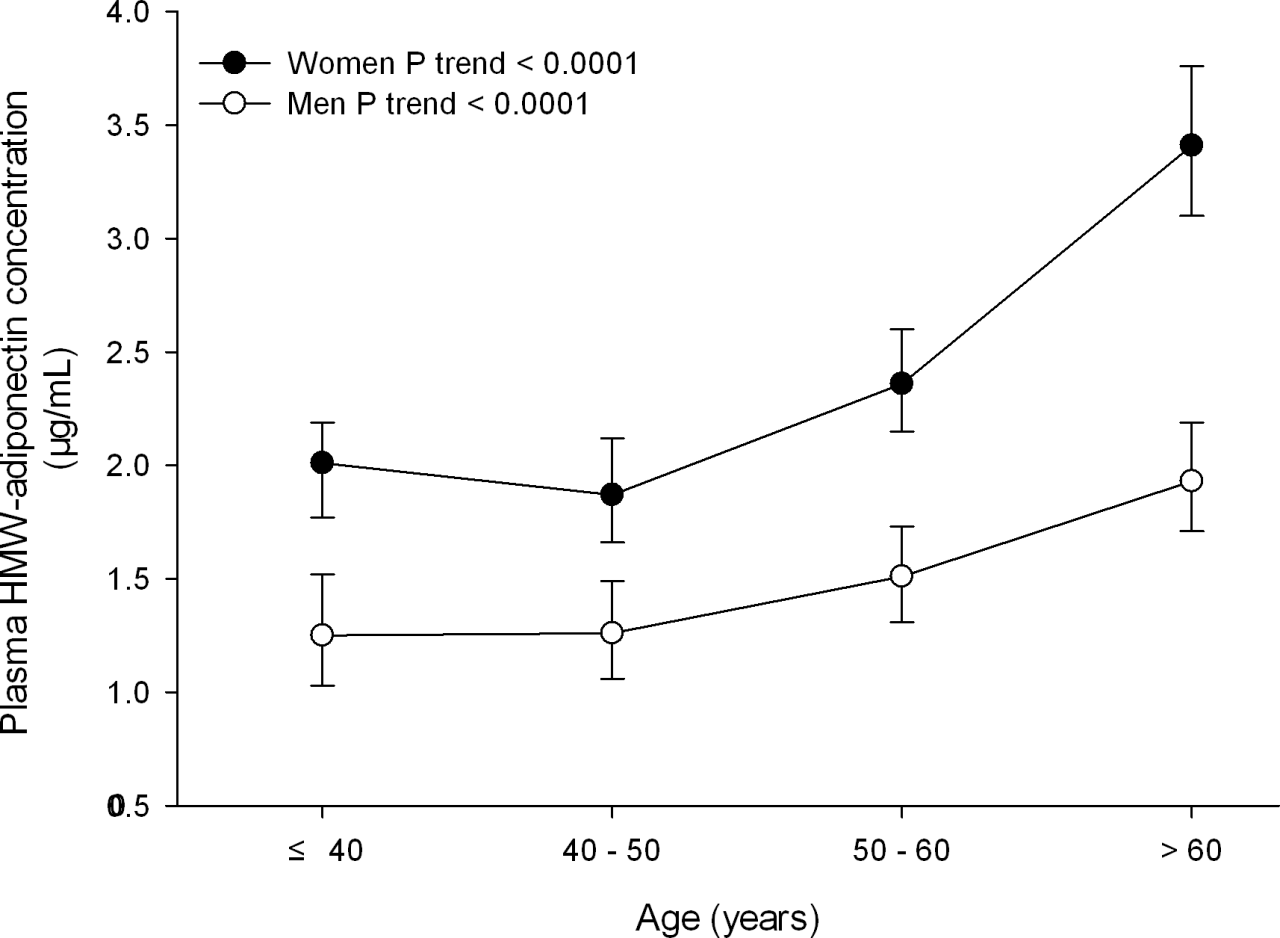
Plasma high molecular weight (HMW) adiponectin concentration by gender and age. Open and closed symbols represent geometric mean of plasma HMW adiponectin concentration in men and women, respectively. Vertical lines denote 95% confidence intervals. The number of subjects for each subgroup is given alongside the symbols. *P* values for trend are given for men and women separately.

Plasma HMW adiponectin concentration was significantly (*P* < 0.0001) higher in women than men (2.47 *vs* 1.58 μg/mL) and increased with advancing age in men (*r* = 0.28) as well as women (*r* = 0.29, **Fig 1**).

In analyses stratified for gender and adjusted for age, current smoking and alcohol intake, the use of antihypertensive medication and the use of antidiabetic drugs, plasma HMW adiponectin concentration was lower in the presence of overweight (n = 159, 1.26 μg/mL in men and n = 227, 2.15 μg/mL in women) and obesity (n = 60, 1.31 μg/mL in men and n = 82, 2.10 μg/mL in women) than normal weight subjects (n = 185, 2.07 μg/mL in men and n = 368, 2.94 μg/mL in women) and in the presence of central obesity (n = 106, 1.28 μg/mL in men and n = 331, 2.12 μg/mL in women) than subjects with a normal waist circumference (n = 298, 1.74 μg/mL in men and n = 346, 2.74 μg/mL in women, **Table 2**). Further stratification for age-subgroup (19–49, 50–59, 60–69, and 70–86 years) in men and women did not materially alter the results (**Table 2**).

**Table 2:** Mean concentration of plasma high-molecular-weight (HMW) adiponectin by gender and obesity.

		Geometric mean concentration of plasma HMW adiponectin (95% confidence interval), μg/mL				
		Body mass index (kg/m^2^)			Waist circumference (cm)	
		<24 (n = 553)	24–27.4 (n = 386)	≥27.5 (n = 142)	<90 cm in men and <80 cm in women (n = 644)	≥90 cm in men and ≥80 cm in women (n = 437)
**Men**		**n = 185**	**n = 159**	**n = 60**	**n = 298**	**n = 106**
	**27–49 years (n = 105)**	**1.52 (1.22–1.91)**	**1.13 (0.92–1.40)**	1.01(0.78–1.32)***	**1.33 (1.13–1.56)**	**1.09(0.88–1.37)**
	**50–59 years (n = 118)**	**2.05 (1.69–2.49)**	1.11 (0.88–1.39)*	**1.37(0.89–2.10)**	**1.67 (1.41–1.98)**	**1.22(0.92–1.62)**^†††^
	**60–69 years (n = 117)**	**1.66 (1.33–2.07)**	**1.23 (1.05–1.54)**	**1.35(0.89–2.04)**	**1.46 (1.25–1.71)**	**1.30(0.89–1.72)**
	**70–86 years (n = 64)**	**3.71 (2.94–4.68)**	2.06 (1.38–3.09)**	1.71(1.01–2.91)**	**3.36 (2.76–4.10)**	**1.77(1.12–2.81)**^†††^
	**All men (n = 404)**	**2.07 (1.86–2.30)**	1.26 (1.12–1.41)*	1.31 (1.08–1.59)*	**1.74 (1.59–1.90)**	**1.28 (1.11–1.48)**^†^
**Women**		**n = 368**	**n = 227**	**n = 82**	**n = 346**	**n = 331**
	**19–49 years (n = 217)**	**2.17 (1.92–2.45)**	1.68 (1.41–2.00)***	**1.77 (1.39–2.25)**	**2.12 (1.89–2.38)**	**1.62 (1.36–1.92)**^††^
	**50–59 years (n = 230)**	**2.88 (2.48–3.35)**	2.03 (1.77–2.33)*	2.06 (1.66–2.54)**	**2.71 (2.37–3.11)**	**2.00 (1.76–2.27)**^††^
	**60–69 years (n = 140)**	**3.83 (3.12–4.71)**	2.67 (2.28–3.13)**	2.43 (1.96–3.00)**	**3.61 (2.98–4.37)**	**2.59 (2.25–2.97)** ^††^
	**70–84 years (n = 90)**	**4.86 (3.90–6.01)**	**3.57 (2.91–4.38)**	**3.63 (2.85–4.63)**	**4.23 (3.43–5.21)**	**3.70 (3.13–4.60)**
	**All women (n = 677)**	**2.94 (2.72–3.18)**	2.15 (1.98–2.33)*	2.10 (1.87–2.35)*	**2.74 (2.53–2.97)**	**2.12 (1.97–2.29)**^†^

The analysis was adjusted for current smoking and alcohol intake, the use of antihypertensive medication and the use of antidiabetic drugs in gender- and age-subgroups and additionally for age in all men and women, respectively.

*P < 0.001

**P < 0.01 and

***P < 0.05 versus subjects with a body mass index <24 kg/m².

^†^*P* < 0.001

^††^*P* < 0.01 and

^†††^*P* < 0.05 versus subjects with a waist circumference <90 cm in men and <80 cm in women.

In multiple regression analyses stratified for gender and adjusted for age, current smoking and alcohol intake, the use of antihypertensive medication and the use of antidiabetic drugs, we investigated the relationship between the closely correlated (r 0.43 to 0.98, *P* < 0.001, **Table 3**) anthropometric and BIA measures of adiposity and plasma adiponectin concentration. With each of the adiposity measures considered separately all these anthropometric and BIA measures were significantly (*r* -0.18 to -0.31, *P* < 0.001) and negatively associated with plasma HMW adiponectin concentration in men as well as women (**Table 4**). However, in further multiple stepwise regression analysis, only waist-to-hip ratio entered and stayed in the model (*P* < 0.05) in men (*r* = -0.10) and women (*r* = -0.15, **Table 4**).

**Table 3:** Pearson’s correlation coefficients between anthropometric and bioelectrical impedance analysis measures of adiposity.

		Body mass index (kg/m^2^)	Waist circumference (cm)	Waist-to-hip ratio	Body fat percentage (%)	Visceral fat percentage (%)	Visceral-body fat ratio
**Men**							
	**Body weight (kg)**	**0.88**	**0.85**	**0.53**	**0.43**	**0.84**	**0.80**
	**Body mass index (kg/m**^**2**^**)**		**0.85**	**0.60**	**0.55**	**0.98**	**0.91**
	**Waist circumference (cm)**			**0.83**	**0.56**	**0.83**	**0.74**
	**Waist-to-hip ratio**				**0.53**	**0.59**	**0.48**
	**Body fat percentage (%)**					**0.55**	**0.22**
	**Visceral fat percentage (%)**						**0.92**
**Women**							
	**Body weight (kg)**	**0.90**	**0.84**	**0.44**	**0.60**	**0.84**	**0.84**
	**Body mass index (kg/m**^**2**^**)**		**0.85**	**0.50**	**0.71**	**0.97**	**0.96**
	**Waist circumference (cm)**			**0.77**	**0.66**	**0.83**	**0.83**
	**Waist-to-hip ratio**				**0.44**	**0.48**	**0.47**
	**Body fat percentage (%)**					**0.73**	**0.61**
	**Visceral fat percentage (%)**						**0.97**

*P* < 0.001 for all correlation coefficients.

**Table 4:** Association of plasma high-molecular weight adiponectin concentration with various adiposity measures by gender.

		Men (n = 404)		Women (n = 677)	
		Considered separately^#^	Considered stepwise^†^	Considered separatedy^#^	Considered stepwise ^†^
**Anthropometric measurements**					
	**Body weight**	-0.29*	**NS**	-0.20*	**NS**
	**Body mass index**	-0.31*	**NS**	-0.25*	**NS**
	**Waist circumference**	-0.28*	**NS**	-0.24*	**NS**
	**Waist-to-hip ratio**	-0.26*	-0.10**	-0.26*	-0.15*
**Bioelectrical impedance analysis measurements**					
	**Body fat percentage**	-0.18*	**NS**	-0.21*	**NS**
	**Visceral fat percentage**	-0.31*	**NS**	-0.24*	**NS**
	**Visceral-body fat ratio**	-0.29*	**NS**	-0.25*	**NS**

Values are Pearson partial correlation coefficient generated in multiple regression analyses.

^**#**^Multiple regression analyses adjusted for age, current smoking and alcohol intake, the use of antihypertensive medication and the use of antidiabetic drugs and considered separately the measures of adiposity.

^**†**^Multiple regression analyses adjusted for age, current smoking and alcohol intake, the use of antihypertensive medication and the use of antidiabetic drugs and considered stepwise the measures of adiposity.

*P < 0.001

**P < 0.05.

**Fig 2:**
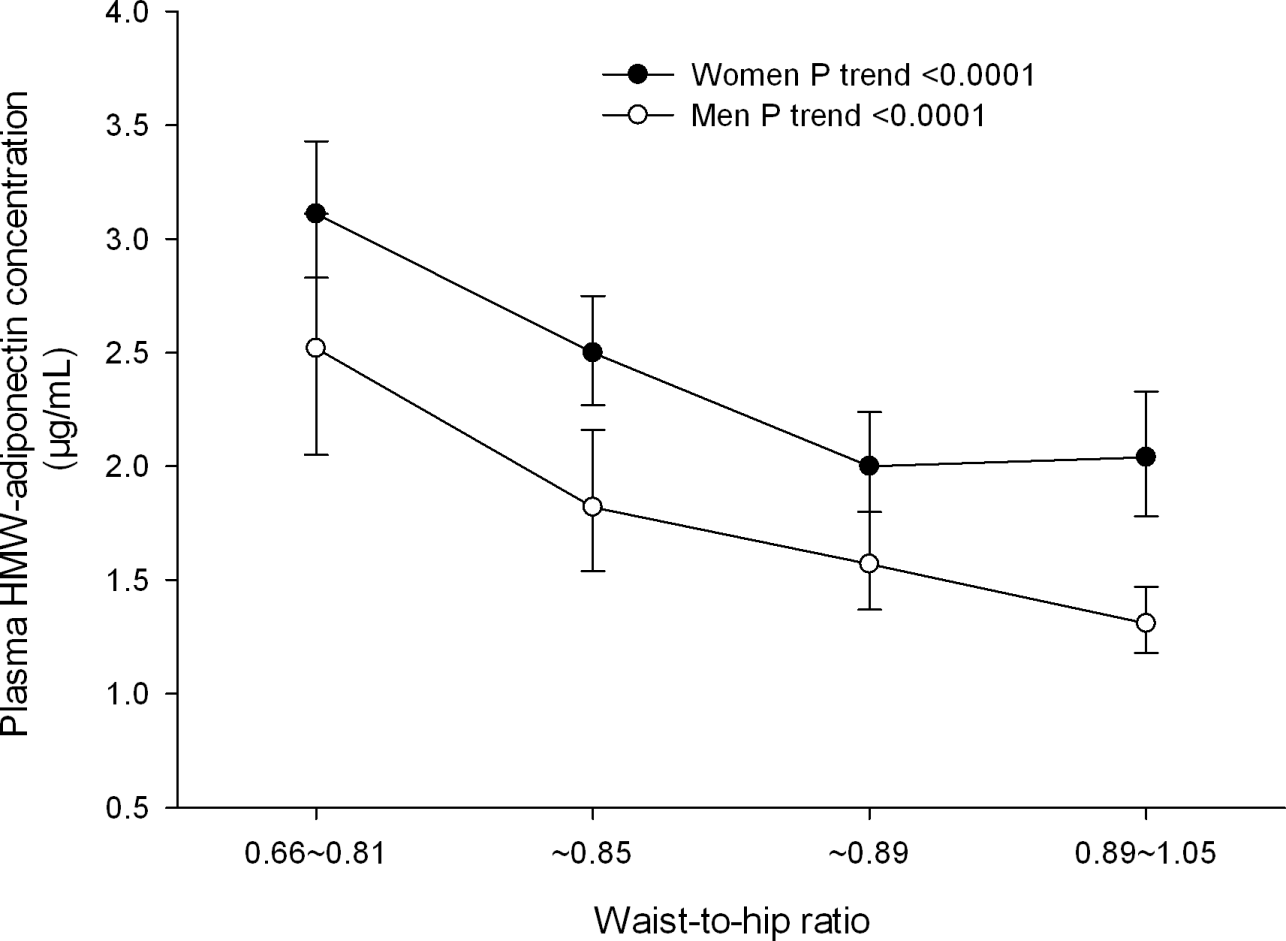
Plasma high molecular weight (HMW) adiponectin concentration by gender and quartile distributions of waist-to-hip ratio. Open and closed symbols represent geometric mean value of plasma HMW adiponectin concentration in men and women, respectively. Vertical lines denote 95% confidence intervals. *P* values for trend are given for men and women separately.

In further analysis of covariance adjusted for age, current smoking and alcohol intake, the use of antihypertensive medication and the use of antidiabetic drugs, subjects were categorized according to gender and quartiles of waist-to-hip ratio, plasma HMW adiponectin concentration was 62.1% and 61.7% lower in the top versus bottom quartiles of waist-to-hip ratio in men and women, respectively (*P* < 0.0001, **Fig 2**). In multiple logistic regression analyses adjusted for age, current smoking and alcohol intake, the use of antihypertensive medication and the use of antidiabetic drugs, the corresponding odds ratios of hypoadiponectinemia were 1.58 (95% CI 0.62–4.02) and 2.81 (95% CI 1.22–6.49), respectively.

## Discussion

The key finding of our study is that waist-to-hip ratio, but not the BIA adiposity measure, is independently associated with plasma HMW adiponectin concentration, which is substantially lower in the presence of overweight, obesity or central obesity. A possible implication of our observation is that a simple measure of central obesity, waist-to-hip ratio, can be even more useful than the device-based BIA measure, in terms of the association with adiponectin concentration.

Plasma adiponectin is a well-proven and recognized marker of adiposity [22]. Indeed, plasma adiponectin concentration was lower in the presence of obesity [23,24], irrespective of obesity pattern [23], study population, or whether it was measured as total or HMW form [24]. There is consensus that hypoadiponectinemia is mainly, if not solely, related to visceral obesity, as either effect or cause. Our cross-sectional study does not have the possibility to draw any conclusion on causality, but does support an independent relationship between plasma adiponectin concentration and visceral obesity. Nonetheless, using a Mendelian randomization approach, a recent study did not support a causal relationship between reduced circulating adiponectin levels and two obesity-related metabolic disorders, i.e., insulin sensitivity and type 2 diabetes mellitus.

A surprising observation is that in multiple stepwise regression analyses, waist-to-hip ratio, but not the BIA visceral fat index, was significantly associated with plasma adiponectin concentration. This novel observation is not entirely understood. There are several speculative explanations. First, algorithms used in BIA devices involve many other factors in addition to the measured impedance values for the computation of visceral fat index. These multiple factors may increase random errors and decrease accuracy of measurement [17, 25]. Second, the device-based indexes do not properly account for percutaneous fat, as waist-to-hip ratio does. The visceral-body fat ratio seemed to have quantitatively improved correlation with plasma adiponectin concentration, but was still far below waist-to-hip ratio, and was not statistically significant.

It is also possible that the two-limb and four-limb BIA devices, though validated against the CT or MRI gold standard [17], are not sufficiently accurate in the measurement of central obesity. In attempt to improve accuracy of measurement, BIA devices with multiple local abdominal electrodes recently became available [25]. Whether these complex abdominal BIA devices would be better or do better than the limb BIA devices and anthropometric measurements remains to be elucidated.

Our finding is actually in keeping with a recent study in 1056 adult Yup’ik people from 11 communities in Southwestern Alaska, USA [26]. Anthropometric and BIA measures of adiposity were correlated with various biomarkers of obesity and metabolism, including adiponectin. Multiple regression analyses were not performed in this Alaskan study. However, univariate analyses revealed that the correlation coefficients for anthropometric measurements, such as waist circumference (r = -0.43), were numerically greater than those for foot-to-foot BIA measures of adiposity, such as fat mass (r = -0.41) [26]. The investigators of the Alaskan study therefore concluded that waist circumference and other anthropometric measures were more closely correlated with obesity-related risk factors than BIA estimates of body composition, and that body composition in Yup’ik people could be accurately estimated from simple anthropometrics. The consistency between our finding and the results of the Alaskan study corroborates the use of simple anthropometric measures in the evaluation of adiposity.

Our study should be interpreted within the context of its limitations. First, we chose a biochemical marker of obesity to compare anthropometric and BIA measurements. The observed inferiority of BIA obesity measures in association with plasma adiponectin concentration does not exclude diagnostic or prognostic values of BIA obesity measures for other metabolic disorders. Second, because the more professional BIA devices with multiple abdominal electrodes only became available recently [25], we used a four-limb device for BIA measurements. Third, because of missing data, we excluded from the present analysis approximately 10% of the study participants. This approach may be potentially a source of selection bias. However, we do not have a better alternative other than excluding the subject with missing data. Fourth, the participation rate of our study was 73.1%. There was therefore some possible selection bias.

In conclusion, anthropometric measures of obesity, such as waist-to-hip ratio, but not BIA measures, obtained with a four-limb device, are independently associated with plasma adiponectin. In spite of the increasing interest in the use of simple or complex BIA devices for the evaluation of obesity, a simple anthropometric measure, such as waist-to-hip ratio, might still be useful or irreplaceable in a country with a vast number of visceral obese people, such as China.

## Supporting Information

S1 File This file contains the participant-level data. (SAS7BDAT)
